# Leaders’ achievement goals predict employee burnout above and beyond employees’ own achievement goals

**DOI:** 10.1111/jopy.12427

**Published:** 2018-09-04

**Authors:** Roy B. L. Sijbom, Jonas W. B. Lang, Frederik Anseel

**Affiliations:** ^1^ Department of Work and Organizational Psychology University of Amsterdam Amsterdam The Netherlands; ^2^ Department of Personnel Management, Work and Organizational Psychology Ghent University Ghent Belgium; ^3^ King’s College London King’s Business School London United Kingdom

**Keywords:** burnout, goal orientation, leadership, motivation, multilevel

## Abstract

**Objective:**

Burnout has primarily been examined from an individual's perspective without taking the broader environmental context into account. The authors applied an integrative, multilevel perspective and investigated the influence of leaders’ motivational strivings on employee burnout. In two multisource studies, we investigated relationships between leaders’ achievement goals and employee burnout while controlling for employees’ own achievement goals.

**Method:**

Study 1 consisted of 362 members and 72 leaders of the corresponding working groups. Study 2 consisted of 177 employees and 46 leaders of the corresponding working groups, and measurements were spaced apart in time. We also ran a model including the data of both Study 1 and Study 2.

**Results:**

Multilevel analyses indicated that leaders’ mastery‐approach goals were negatively related to employee burnout above and beyond employees’ own achievement goals. Leaders’ performance‐approach goals were positively related to employee burnout in Study 1 and in the overall analysis combining Study 1 and Study 2.

**Conclusions:**

We advance our understanding of the motivational etiology of burnout by examining the top‐down effects of leaders’ achievement goals on employee burnout over and above employees’ own achievement goals. In order to reduce burnout, organizations should take leaders’ achievement goals into account as an important contextual factor.

## 1 INTRODUCTION

According to the American Psychological Association (2009), 69% of employees report work as a significant source of job stress. Given that 46% of all employees are severely stressed to the point of burnout (Arnetz, 2006), it might not be surprising that organizations seek to reduce and prevent burnout to avoid potential burnout‐related problems like financial losses, accidents, health problems, reduced productivity, or absenteeism (Maslach, Schaufeli, & Leiter, 2001).

Research on burnout has traditionally focused on understanding how individual‐level factors like personality or perceptions of the work environment are related to burnout (for an overview, see Maslach et al., 2001). Acknowledging the ubiquitous influence that motivational factors have on effectively pursuing or realizing one's goals at work, researchers also started investigating the role of achievement goals, as a motivational disposition, in predicting burnout (Naidoo et al., 2012; Poortvliet, Anseel, & Theuwis, 2015; Tuominen‐Soini, Salmela‐Aro, & Niemivirta, 2008). In this emerging line of research, the influence of achievement goals on burnout has primarily been examined from an individual's perspective, independently of the broader environmental context. This perspective, however, risks painting an incomplete picture of the motivational underpinnings of burnout because, since its origins, it has frequently been emphasized that the emergence of burnout can only be understood by taking the effects of both important work environment characteristics and individual characteristics into account (Leiter & Maslach, 1988). In fact, the burnout literature has responded to a broader trend toward contextualization in the organizational stress literature and has started to adopt an integrative, multilevel perspective (Bliese & Jex, 2002; Ganster & Rosen, 2013; Kozlowski & Klein, 2000).

One important idea in the multilevel literature, which also frequently forms a critical test of the multilevel perspective (Bliese & Jex, 2002; Hofmann & Gavin, 1998), is that environmental factors meaningfully contribute to the explanation of individual‐level outcomes beyond individual‐level perceptions. Such a combined test of individual and situational motivational antecedents of burnout is important, as it would significantly advance our understanding of the complicated motivational etiology of burnout. Theoretically, we need to elucidate how, next to individual motivational strivings, motivational cues in the organizational environment may also provoke burnout and provide an initial test of their relative strength in doing so. From a practical point of view, this may also offer an alternative line of preventive interventions that not only target maladaptive tendencies in the individual, but that may also consider potential beneficial or detrimental motivational cues in the environment. Therefore, in the present research, we build on these ideas and investigate leaders’ achievement goals as a contextual predictor of employee burnout above and beyond employees’ personal achievement goals. Thus, we aim to extend the dispositional perspective on burnout by looking not only at individuals’ trait motivation as a predictor, but also their leaders’ trait motivation. In doing so, we investigate the influence of higher‐level variables (leaders’ achievement goals) on lower‐level variables (employee burnout).

In a work context, leaders—more specifically, leaders’ achievement goals—provide an important and dominant source of social cues for their employees (Chen & Bliese, 2002; Dragoni, 2005; Dweck & Leggett, 1988). Drawing on achievement goal theory (Elliot & McGregor, 2001), we argue that the way in which leaders interpret, approach, and respond to achievement situations will result in leaders’ displaying different behaviors (cf. Dragoni, 2005). We theorize that this pattern of leader behavior, which reflects the leader's achievement priority, influences employee burnout by providing supportive resources for employees (e.g., focus on learning) or imposing demands (e.g., competition with others) that may deplete employees’ resources. At this point, it is unclear how much leader achievement motivation vis‐à‐vis individual achievement motivation may provide or deplete resources to eventually affect the risk of burnout. As such, the aim of this article is to investigate the incremental validity of leaders’ achievement goals above and beyond employees’ own achievement goals in predicting burnout using a multilevel perspective. Specifically, we suggest that leaders’ mastery‐approach goals are negatively related to employee burnout and leaders’ performance‐approach goals are positively related to employee burnout.

We critically test our theoretical ideas in two different multisource, multilevel field studies, with achievement goal and burnout measures separated in time in the second study. Multiple studies allow for a better test of generalized causal inference by employing diverse methodologies (Shadish, Cook, & Campbell, 2002). In the first study, we test a multisource, cross‐sectional design using 362 employees from 72 working groups and the 72 leaders of these working groups. In the second study, we use a design in which the individual‐level measurements were spaced apart in time using 177 employees from 46 working groups and the 46 leaders of these working groups. In both studies, we are linking leader reports of their own achievement goals with employee reports of burnout to avoid common method bias.

### 1.1 Burnout

Burnout is conceived of as a three‐dimensional concept that consists of emotional exhaustion, cynicism, and reduced professional efficacy (Maslach et al., 2001; Schaufeli, Leiter, Maslach, & Jackson, 1996). Emotional exhaustion represents a basic stress response and occurs when individuals’ emotional resources are depleted. Cynicism refers to negative feelings and cynical attitudes about one's work. The third dimension, reduced professional efficacy, involves the degrading of one's belief that one can effectively complete one's work (Maslach et al., 2001).

Although initially developed as a three‐dimensional construct (Maslach, 1982), there is an ongoing debate among researchers whether burnout is best conceptualized by two dimensions, whereby reduced professional efficacy is not considered as a component of burnout (Demerouti, Bakker, Nachreiner, & Schaufeli, 2001). Accumulating evidence indicates that reduced professional efficacy plays a divergent role as compared to emotional exhaustion and cynicism (e.g., Lee & Ashforth, 1996), which are considered to be the “core” dimensions of burnout (Schaufeli & Taris, 2005). However, the contemporary evidence is mixed, and to date there exists no consensus regarding whether burnout is best conceptualized as a two‐ or three‐dimensional construct. Therefore, in this article, we remain consistent with the original three‐dimensional conceptualization of burnout.

### 1.2 Achievement goals

Achievement goals represent competence‐relevant aims that individuals adopt and pursue in achievement situations, including the workplace (DeShon & Gillespie, 2005; Elliot, 2005). In the conceptualization of achievement goals, the focus on an interpersonal standard (i.e., others) is referred to as a performance goal, whereas the focus on an intrapersonal standard (i.e., the self) is referred to as a mastery goal. In Elliot and McGregor's (2001) 2 × 2 framework, a further distinction is made between approach goals, aimed at acquiring positive possibilities, and avoidance goals, aimed at avoiding negative possibilities (Elliot, 1999). This results in four distinctive goals of the 2 × 2 achievement goal framework: mastery‐approach goals, which entail striving to do better than before; mastery‐avoidance goals, which entail striving to avoid doing worse than one has done before; performance‐approach goals, which entail striving to do better than others; and performance‐avoidance goals, which entail striving to not do worse than others (Elliot, 2005; Van Yperen & Orehek, 2013). Although an individual may hold multiple achievement goals (i.e., an achievement goal profile; Tuominen‐Soini, Salmela‐Aro, & Niemivirta, 2012; Van Yperen & Orehek, 2013), our interest here is on the strength of any one kind of achievement goal.

Conceptual and empirical considerations suggest that achievement goals may be best suited for the domain‐specific level rather than the dispositional‐specific level (cf. Baranik, Barron, & Finney, 2010; Elliot, 2005). Domain‐specific achievement goals refer to stable patterns of cognition and action that result from the pursuit of achievement goals in a specific situation, whereas dispositional achievement goals reflect a similar pattern in different situations over time (DeShon & Gillespie, 2005). In line with prior research and following the conceptualization of Elliot (2005), we examined leaders’ personally adopted achievement goals from a domain‐specific perspective. That is, in our study, we focus on the effects of stable leaders’ achievement goals specific to the work domain.

### 1.3 Leaders’ achievement goals and employee burnout

In the execution of daily work tasks, employees may face both successes and setbacks. Setbacks may lead to frustrated goal striving when employees do not have adequate efficient strategies or coping mechanisms. Achievement goals may be relevant here because the striving of specific goals significantly affects not only how and why employees are motivated to execute their work tasks, but also how they deal with setbacks and frustrated goal striving. This latter aspect is especially relevant for studying burnout because frustration in the execution of goal striving and a lack of efficient strategies to cope with it is a potent source for the development of burnout. Accumulated evidence indicates that at the individual level, achievement goals are an important antecedent of employee burnout (Naidoo et al., 2012; Retelsdorf, Butler, Streblow, & Schiefele, 2010). Individuals with high mastery‐approach goals have efficient strategies and coping mechanisms to deal with stressful situations and frustrated goal striving (Payne, Youngcourt, & Beaubien, 2007; Rawsthorne & Elliot, 1999). For them, setbacks and failure are perceived as diagnostic information that signifies potential for self‐improvement. This focus on self‐improvement is instrumental in developing and learning new ways to handle challenges. In contrast, individuals pursuing high performance‐approach goals may perceive setbacks and failures as evaluative information that frustrates their goal of demonstrating competence to others, resulting in maladaptive response patterns, such as effort withdrawal, helplessness, anxiety, low persistence, or even complete withdrawal from the task (Butler, 1993; Dweck & Leggett, 1988; Nicholls, 1984; VandeWalle, Cron, & Slocum, 2001). Furthermore, mastery‐avoidance goals and performance‐avoidance goals have been reported to show an underlying fear of failure (Elliot & McGregor, 2001). Fear of failure may drain a person's energy resources (Demerouti et al., 2001), making a person less able to cope with stress resulting from frustrated goal striving. Taken together, previous research at individual‐level factors consistently showed that achievement goals are related to the coping mechanisms and strategies that individuals employ when faced with setbacks and frustrated goal strivings, which, in turn, affect whether they experience burnout.

The burnout literature emphasized that the emergence of burnout can only be understood by taking the effects of both important work environment characteristics and individual characteristics into account (Leiter & Maslach, 1988). Indeed, virtually all stress models delineate that problematic stress experiences at work originate at the interface of environment and individual (Ganster & Rosen, 2013). In a work context, leaders, and more specifically, leaders’ achievement goals, provide an important and dominant source of social cues for their employees (Chen & Bliese, 2002; Dragoni, 2005; Dweck & Leggett, 1988). We argue that leaders’ achievement goals may predict employee burnout above and beyond employees’ own achievement goals. The theoretical rationale underlying the expected multilevel main effects of leaders’ achievement goals (higher‐level variable) on employee burnout (lower‐level variable) is based on the idea that leaders’ achievement goals result in corresponding patterns of leader behavior that may provide supportive resources for employees or impose demands that may consume resources. A primary responsibility of leaders is to provide resources (e.g., social support, feedback, growth opportunities) so that employees can successfully complete work (Chemers, 2000). As resources minimize the impact of work‐related stressors, they protect individuals from negative consequences of stress (e.g., burnout). The resources leaders provide may be driven by the achievement goals these leaders pursue. That is, leaders transmit their achievement goal priority by engaging in behaviors and practices that support, reinforce, and imply their favored achievement goal (e.g., Ames & Archer, 1988; Dragoni, 2005; Dweck & Leggett, 1988). These behaviors and practices, in turn, may (not) be helpful for employees in dealing with setbacks and frustrated goal striving.

### 1.4 Leaders’ mastery‐approach goals and employee burnout

Leaders with high mastery‐approach goals typically prioritize personal effort, learning, and experimentation, stressing that trying hard and individual improvement are valued, and that experiencing failure is part of the learning process (Blecharz, Luszczynska, Tenenbaum, Scholz, & Cieslak, 2014; Dragoni, 2005; Lau & Nie, 2008). These leaders are also likely to emphasize acquisition of skills and offer feedback that promotes employees’ efficacy in attaining goals (cf. Kozlowski, Gully, Salas, & Cannon‐Bowers, 1996). Moreover, leaders pursuing mastery‐approach goals may employ different management strategies to convey their dedication to learning: offering time off to participate in developmental activities (Maurer & Tarulli, 1994), and advising employees to implement newly learned skills on the job (Ford, Quinones, Sego, & Sorra, 1992). Together, these management practices and behaviors are characterized by emphasizing self‐improvement features like learning, allowing failure, and encouraging experimentation, which may provide employees with resources in dealing with setbacks. For example, employees may be more inclined to experiment with new strategies to cope with frustrated goal striving. Also, when leaders emphasize that encountering failure is part of the learning process, employees may be more comfortable when they experience failure during the execution of their work. Rather than perceiving this failure as a stressful situation that consumes resources, employees may perceive it as a way that helps them to learn and develop. Failure may thus be a supportive resource because it may be perceived as useful diagnostic feedback information that helps them to achieve their work goal. Accordingly, we hypothesize the following:

Hypothesis 1 A higher mastery‐approach goal in leaders is negatively related to employee burnout above and beyond the effects of employees’ own achievement goals.

### 1.5 Leaders’ performance‐approach goals and employee burnout

Leaders with high performance‐approach goals are focused on competence demonstration relative to others (Dweck & Leggett, 1988). These leaders prioritize demonstration of ability in which employees are encouraged to engage in competition with one other for the receipt of extrinsic rewards. As such, these leaders establish social cues in the work environment in which interpersonal competition, outperforming others, and the demonstration of ability prevail (Ames, 1992; Dragoni, 2005). By explicitly comparing the performance of employees relative to others, performance‐approach goal leaders may (implicitly) encourage impression management techniques among employees. Also, prioritizing the demonstration of ability may lead to self‐presentation concerns among employees that can be very stressful and resource consuming. Rather than providing supporting resources, these management practices and behaviors may impose demands on employees that consume resources. For example, when faced with setbacks and frustrated goal striving, employees may be concerned about their public image because their task performance will be compared relative to others. This focus on self‐presentation concerns may cause evaluation stress among employees, which may lead to increased feelings of burnout. Likewise, when leaders emphasize that failure is a signal of incompetence and inability, employees may become stressed when they are faced with setbacks or frustrated goal striving. Because of the salience of normative aspects, frustrated goal striving may result in a fear of appearing incompetent. Accordingly, employees may perceive such situations as stressful. Given that dealing with these stressful situations consumes (cognitive) resources, it makes them more prone to burnout. Finally, emphasis on normative comparison by the leader may even turn positive resources (e.g., successfully accomplishing a task) into demands (e.g., the completion is interpreted as inferior because others accomplished a similar task in less time) for employees. Accordingly, we hypothesize the following:

Hypothesis 2 A higher performance‐approach goal in leaders is positively related to employee burnout above and beyond the effects of employees’ own achievement goals.

We expect that higher mastery‐approach goals and higher performance‐approach goals in leaders will explain variance in employee burnout over and above employees’ own achievement goals. In contrast, leaders with avoidance goals (either mastery‐avoidance or performance‐avoidance) have an inherently negative focus on their work‐related efforts and may feel depleted and unable to engage in (proactive) behaviors to help employees attain their goals (cf. Oertig et al., 2013), such as establishing rewarding social relationships with employees in the working environment. Moreover, avoidance strivings in leaders may undermine their own motivation to act (cf. Ali, Ryan, Lyons, Ehrhart, & Wessel, 2016), meaning that they are neither a source of resources nor a source of demands for their employees. Because avoidance strivings in leaders (either mastery‐avoidance or performance‐avoidance) seem related to withdrawal from influence processes, we did not anticipate any effects of leader avoidance goals on employee burnout. However, we exploratively examined possible relationships for the sake of completeness.

## 2 METHOD

We tested our hypotheses in two separate samples (Study 1 and Study 2). We describe the samples and procedures of each study below.

### 2.1 Sample and procedure: Study 1

Our sample consisted of working groups representing several industries from Belgian organizations, including education, retailing, manufacturing, banking, police, and medical service, among others. Our multi‐industry focus was intentional, as sampling diversity across industries helps avoid contextual constraints associated with any particular organization type (cf. Yang, Mossholder, & Peng, 2007). The sample was collected by student groups (4–5 students per group) as part of a work and organizational psychology course given by the first author. Each of the 24 student groups collected data from three working groups (each working group consisting of a leader and five employees). Students were instructed to use their own personal network (i.e., family, friends, etc.) to recruit potential participants, either directly (i.e., contacting individuals in supervisory positions) or indirectly (i.e., students’ personal contacts inquired whether other employees and the direct supervisor of their working group were willing to participate; student‐recruited sampling; Demerouti & Rispens, 2014). The students emphasized that participation of the supervisor and the group members was voluntary. A survey package containing one supervisor survey, five employee surveys, and six envelopes was given to the supervisor (some supervisors received seven employee surveys on their own request), who distributed the questionnaires to the employees within his or her working group. On the first page of each questionnaire, a cover letter explained the nature of the research and assured participants of anonymity and confidentiality. The supervisor of each work group was instructed by the students to inquire about subordinates’ interest in the survey to ensure voluntary participation.

Survey participants consisted of 372 employees and 72 supervisors of 72 working groups. Two employee respondents did not hand in their questionnaire to their supervisor, and eight employee respondents had more than 50% missing data or completely missing data on the study variables. The final sample consisted of 362 employees nested in 72 groups. Group sizes ranged from 3 to 7 (*M* = 5.03, *SD* = 0.50). Of the employees responding, 41% were men, and 72% had a college degree or higher. Their ages ranged from 18 to 66 years, with a mean age of 38.3 years (*SD* = 10.8). Seventy‐nine percent of the employees had a full‐time position, and mean organizational tenure in the current organization was 11.3 years (*SD* = 9.9). Sixty‐six percent of the supervisors were men, and 72% had a college degree or higher. Their mean age was 46.0 years (*SD* = 9.1), and mean organizational tenure was 24.1 years (*SD* = 8.7).

### 2.2 Sample and procedure: Study 2

We tested our hypotheses in a sample of work groups representing several industries from Dutch organizations. Data were collected by trained students as part of a work psychology research project under the first author's supervision. The students contacted leaders working in organizations regarding their groups’ interest in participating. The supervisors provided their email addresses and those of 5–10 employees (although more could be provided if desired), to whom an online survey was sent. At the start of each survey, a cover letter explained the nature of the research and assured participating individuals of anonymity and confidentiality.

At Time 1, online surveys were sent to 710 employees and 106 supervisors of 106 working groups. We received 390 employee questionnaires and 86 supervisor questionnaires that could be used (T1 employee response rate = 54.9%; supervisor response rate = 81.1%). At Time 2, three months later, we sent the questionnaires to the 390 employees who completed the Time 1 questionnaires. We received 243 employee questionnaires that could be used (T2 employee response rate = 62.3%). We included only teams for whom we received two or more employee surveys. Our final sample contained 177 employees and 46 supervisors of 46 working groups who completed all relevant study variables at T1 and T2, meaning an overall employee response rate of 24.9% and a supervisor response rate of 43.4%.

Group sizes ranged from 2 to 13 (*M* = 3.85, *SD* = 2.31). Of the employees responding, 62% were men, and 37% had a college degree or higher. Their ages ranged from 18 to 66 years, with a mean age of 40.8 years (*SD* = 13.6). Eighty‐one percent of the employees had a full‐time position (more than 35 hr/week), and mean organizational tenure in the current organization was 10.5 years (*SD* = 9.5). Sixty‐three percent of the supervisors were men, and 59% had a college degree or higher. Their mean age was 43.5 years (*SD* = 11.8), and mean tenure in the current leadership position was 10.6 years (*SD* = 9.7).

### 2.3 Measures: Study 1 and Study 2

We used the supervisor questionnaire to assess leaders’ achievement goals. Using the employee questionnaire, we measured employees’ achievement goals and burnout. In Study 2, the measurements of employees’ achievement goals and employee burnout were spaced 3 months apart to minimize potential common method effects (see Podsakoff, MacKenzie, Lee, & Podsakoff, 2003).

#### 2.3.1 Leaders’ achievement goals

Leaders’ mastery‐approach goal, leaders’ performance‐approach goal, leaders’ mastery‐avoidance goal, and leaders’ performance‐avoidance goal were measured using the corresponding three‐item subscales of the Achievement Goal Questionnaire‐Revised (AGQ‐R; Elliot & Murayama, 2008). Items were adapted to fit the work context of the research. Our scale modification entailed changing the domain from a class setting (“In my classes”) to a work setting (“In my work”; for similar adaptations, see Sijbom, Janssen, & Van Yperen, 2015; Van Yperen & Janssen, 2002). Participants rated three items for each goal construct: mastery‐approach goal (e.g., “My aim is to perform better in my work than I have done in the past”), performance‐approach goal (e.g., “In my work my goal is to do better than other colleagues”), mastery‐avoidance goal (e.g., “My goal in my work is to avoid doing worse than I have done before”), and performance‐avoidance goal (e.g., “My aim at work is to avoid doing worse than others”). In Study 1, the response categories ranged from 1 (*not true of me*) to 7 (*extremely true of me*), whereas in Study 2, the response categories ranged from 1 (*not true of me*) to 5 (*extremely true of me*).

#### 2.3.2 Employees’ achievement goals

Employees’ mastery‐approach goals, employees’ performance‐approach goals, employees’ mastery‐avoidance goals, and employees’ performance‐avoidance goals were measured using the same corresponding three‐item subscales of Elliot and Murayama's (2008) AGQ‐R and the same response categories as used for measuring leaders’ achievement goals.

#### 2.3.3 Employee burnout

In Study 1 and Study 2, we measured employee burnout with three subscales of the Dutch version of the Maslach Burnout Inventory‐General Survey (MBI‐GS; Schaufeli et al., 1996): Emotional Exhaustion (five items), Cynicism (five items), and Professional Efficacy (six items). In both studies, responses were rated on a 7‐point scale ranging from 1 (*never*) to 7 (*always*). Sample items include “I feel emotionally drained by my work” (Emotional Exhaustion); “I doubt the significance of my work” (Cynicism); and “I feel I am making an effective contribution to what this organization does” (Professional Efficacy). The Professional Efficacy subscale items were reverse coded for the purpose of comparison with the other two subscales and now relates to reduced professional efficacy. Here, we use the overall composite Burnout scale including all items. The MBI‐GS has been psychometrically validated (e.g., Schutte, Toppinen, Kalimo, & Schaufeli, 2000).

### 2.4 Analytical strategy

We used linear mixed effects models (also known as hierarchical linear models; e.g., Raudenbush & Bryk, 2002) to account for the nested data structure (employees nested within leaders). All analyses were conducted using the lme4 package (Bates, Mächler, Bolker, & Walker, 2014) in the statistical software R (R Core Development Team, 2014). We group‐mean‐centered the individual‐level predictors. We used the Satterthwaite approximation *t* test in the lmerTest package to determine the degrees of freedom in testing for significance. We first estimated intraclass correlations type 1 (ICC1s). The ICC1 indicates the percentage of variance in the dependent variable that is explained by group membership. We then fitted the following model including the four employee achievement goals (Level 1 variables) and the four leader achievement goal variables (Level 2 variables) to test our hypotheses^1^:

$$\text{Level}\;1:Y_{\mathrm{ij}}=\beta_{0j}+\beta_{1j}{\text{MAP}}_{\mathrm{ij}}+\beta_{2j}{\text{PAP}}_{\mathrm{ij}}+\beta_{3j}{\text{MAV}}_{\mathrm{ij}}+\beta_{4j}{\text{PAV}}_{\mathrm{ij}}+e_{\mathrm{ij}}$$

$$\text{Level}\;2:\beta_{0j}=\gamma_{00}+\gamma_{01}{\text{LMAP}}_{j}+\gamma_{02}{\text{LPAP}}_{j}+\gamma_{03}{\text{LMAV}}_{j}+\gamma_{04}{\text{LPAV}}_{j}+u_{0j}$$

$$\beta_{1j}=\gamma_{10}+u_{1j}$$

$$\beta_{2j}=\gamma_{20}+u_{2j}$$

$$\beta_{3j}=\gamma_{30}+u_{3j}$$

$$\beta_{4j}=\;\gamma_{40}+u_{4j}$$

In this model specification, MAP, PAP, MAV, and PAV refer to mastery‐approach, performance‐approach, mastery‐avoidance, and performance‐avoidance goals of the followers, respectively. LMAP, LPAP, LMAV, and LPAV are the mastery‐approach, performance‐approach, mastery‐avoidance, and performance‐avoidance goals of the followers’ leader. The variable *i* is an index for the follower and *j* for the leader.

We report the results for Study 1 and Study 2 separately. However, because the two studies shared a similar design and measures, we also ran a model integrating the data of both Study 1 and Study 2. In this model, we controlled for the origin of the data by including a dummy for the site (coded Study 1 = 0, Study 2 = 1) so that the equation for β_0j_ in the model specification shown above was modified to β_0j_ = γ_00_ + γ_01_LMAP_j_ + γ_02_LPAP_j_ + γ_03_LMAV_j_ + γ_04_LPAV_j_ + γ_05_SITE_j_ + *u*_0j_. Because Study 1 and Study 2 used different scale formats (5‐point Likert scales vs. 7‐point Likert scales), we scaled the continuous predictors in the mixed effects models using the percentage of maximal possible (POMP) scores metric (Cohen, Cohen, West, & Aiken, 1999). POMP scores, a linear transformation of any raw metric (e.g., Likert scale scores), are estimated using the formula ((observed‐minimum)/(maximum‐minimum)) × 100 and have a theoretical range from 0 (their minimum) to 100 (their maximum).

## 3 RESULTS

Table 1 presents the descriptive statistics and correlations for all individual‐ and group‐level variables included in the analyses of Study 1 (below the diagonal) and Study 2 (above the diagonal). As shown in Table 1, ICC1 estimates indicated that for Study 1, 19% of the variance in burnout was between‐groups variance, with the remaining 81% residing within groups. For Study 2, also 19% of the variance in burnout was between‐groups variance. Values of this magnitude are typical for organizational research (Bliese, 2000).

**Table 1:** Descriptive statistics for the individual‐ and group‐level variables

Variable		Study 1^a^			Study 2^b^			1	2	3	4	5
		Mean	*SD*	ICC	Mean	*SD*	ICC					
Employees (Level 1)												
	1. Mastery‐approach goal	69.94	17.27	0.06	75.71	19.60	0.28	*(* *0* *.* *8* *2/* *0.8* *2)*	0.24**	0.11	0.10	–0.33**
	2. Performance‐approach goal	58.97	24.08	0.09	55.93	26.32	0.08	0.42**	*(* *0* *.* *8* *2/* *0.8* *3)*	0.08	0.50**	–0.18*
	3. Mastery‐avoidance goal	73.40	21.65	0.02	67.18	28.75	0.17	0.41**	0.35**	*(* *0* *.* *9* *2/* *0.8* *9)*	0.46**	0.03
	4. Performance‐avoidance goal	57.69	27.77	0.07	48.07	32.55	0.13	0.31**	0.67**	0.53**	*(* *0* *.* *9* *5/* *0.9* *3)*	–0.03
	5. Burnout	26.69	12.84	0.19	25.72	11.98	0.19	–0.21**	–0.01	–0.18**	0.00	*(* *0* *.* *8* *7/* *0.8* *7)*
Leaders (Level 2)												
	1. Leader mastery‐approach goal	67.55	18.78		78.26	16.34		*(* *0* *.* *8* *2/* *0.7* *0)*	0.46**	–0.03	0.08	
	2. Leader performance‐approach goal	62.46	22.76		56.34	25.23		0.58**	*(* *0* *.* *8* *9/* *0.7* *6)*	0.36*	0.61**	
	3. Leader mastery‐avoidance goal	67.28	25.66		60.87	29.13		0.28*	0.44**	*(* *0* *.* *9* *2/* *0.9* *0)*	0.70**	
	4. Leader performance‐avoidance goal	54.55	27.93		46.74	29.58		0.41**	0.55**	0.72**	*(* *0* *.* *9* *3/* *0.9* *2)*	

Note

ICC = intraclass correlation type 1. Values on the diagonal represent reliability estimates of Study 1 and Study 2, respectively. All continuous predictors are in percent of maximal possible score (POMP; Cohen et al., 1999) scaling. POMP scores can be relatively straightforwardly interpreted as the percent of the maximum score that is possible on the scale (e.g., when a participant in Study 2 would always indicate 5 on a 5‐point Likert scale, the score would be 100). Zero‐order correlations of Study 1 are presented below the diagonal. Zero‐order correlations of Study 2 are presented above the diagonal.

a Pairwise *n* for individual‐level analyses was 362, *N* (leaders) = 72.

b Pairwise *n* for individual‐level analyses was 177, *N* (leaders) = 46.

* *p* < 0.05. ^**^ *p* < 0.001.

Table 2 provides the mixed effects modeling results. The table includes models with burnout as the criterion and the four employee achievement goals (Level 1 variables) and the four leader achievement goals (Level 2 variables) as predictors. The analyses are shown both separately for the two samples and also in the combined analysis using the data from both samples. We started by inspecting the effects of employee mastery goals on employee burnout. As indicated by Table 2, especially employee mastery goals were negatively associated with burnout. This finding was expected on the basis of the literature.

**Table 2:** Multilevel modeling results predicting employee burnout

Level and variable		Study 1	Study 2	Combined sample
Employees (Level 1)				
	Intercept	31.65** (3.55)	32.30** (5.76)	30.95** (3.03)
	Mastery‐approach goal	–0.15** (0.05)	–0.17** (0.06)	–0.16** (0.04)
	Performance‐approach goal	0.06 (0.04)	–0.02 (0.05)	0.02 (0.03)
	Mastery‐avoidance goal	–0.06 (0.04)	0.00 (0.04)	–0.02 (0.03)
	Performance‐avoidance goal	0.01 (0.04)	0.00 (0.04)	0.01 (0.03)
	Site (0 = Study 1; 1 = Study 2)			1.38 (1.59)
Leaders (Level 2)				
	Leader mastery‐approach goal	–0.13* (0.06)	–0.16* (0.08)	–0.14** (0.05)
	Leader performance‐approach goal	0.12* (0.05)	0.02 (0.06)	0.08* (0.04)
	Leader mastery‐avoidance goal	–0.07 (0.05)	0.10 (0.05)	0.0 (0.04)
	Leader performance‐avoidance goal	0.02 (0.05)	–0.02 (0.06)	–0.00 (0.04)
Additional information				
	–2 log likelihood (REML)	2,830.76	1,353.71	4,199.26

Note

REML = restricted maximum likelihood estimation. Values in parentheses are standard errors; *t* statistics were computed as the ratio of each regression coefficient divided by its standard error. All continuous predictors are in percent of maximal possible score (POMP; Cohen et al., 1999) scaling. The individual‐level goal predictors (Level 1) were group‐mean‐centered.

* *p* < 0.05. ** *p* < 0.01.

We continued by studying Hypothesis 1, which states that a higher mastery‐approach goal in leaders would be negatively related to employee burnout after controlling for the effects of employees’ own achievement goals. As shown in Table 2, the results indicated a significant effect of leader mastery‐approach goals on employee burnout in Study 1, ϒ_10_ = –0.13, *SE* = 0.06, *t*(71.38) = –2.30, *p* < 0.05, in Study 2, ϒ_10_ = –0.16, *SE* = 0.08, *t*(35.90) = –2.05, *p* < 0.05, and in the combined sample, ϒ_10_ = –0.14, *SE* = 0.05, *t*(110.75) = –3.07, *p* < 0.01. These results provide support for Hypothesis 1.

Hypothesis 2 suggested that a higher performance‐approach goal in leaders would be positively related to employee burnout after controlling for the effects of employees’ own achievement goals. Results in Table 2 indicated that in Study 1, a significant effect of leader performance‐approach goal on employee burnout was found, ϒ_10_ = 0.12, *SE* = 0.05, *t*(72.77) = 2.29, *p* < 0.05. In Study 2, the effect was positive but not significant, ϒ_10_ = 0.02, *SE* = 0.06, *t*(39.45) = 0.37. In the combined sample, we found a significant relationship in the hypothesized positive direction, ϒ_10_ = 0.08, *SE* = 0.04, *t*(114.30) = 2.00, *p* < 0.05. Together, these results provided support for Hypothesis 2. Results from Table 2 further show that both leader mastery‐avoidance goals and leader performance‐avoidance goals were not significantly related to employee burnout in neither of the three analyses.

## 4 DISCUSSION

This study was motivated to advance our understanding of the motivational etiology of burnout by examining the cross‐level effects of leaders’ achievement goal strivings on employee burnout. The results of both studies provided evidence indicating that leaders’ mastery‐approach goals and leaders’ performance‐approach goals are related to employee burnout over and above effects of employees’ own achievement goals. Specifically, in both Study 1 and Study 2, and the overall analysis combining the studies, leaders’ mastery‐approach goals were negatively related to employee burnout. We found evidence that leaders’ performance‐approach goals were positively related to employee burnout in both Study 1 and the combined studies, but not in Study 2. We believe these results are particularly compelling, as they were obtained in the context of analytic models that included employees’ own achievement goals, thereby signaling the importance of leader achievement goals, as a higher‐level variable, in predicting employee burnout. Together, these results provide an important contribution to one of the key tenets of the burnout literature. Burnout researchers have long hypothesized that the emergence of burnout should best be understood by taking effects of both important contextual and individual factors into account (Leiter & Maslach, 1988). However, notwithstanding these calls, burnout studies have often failed to adequately model an integrative, multilevel perspective.This oversight in the literature is understandable given the difficulty of obtaining multilevel field samples and because of the appropriate statistical analyses’ not being sufficiently developed in the early days of burnout research. However, by demonstrating cross‐level influences of leaders’ mastery‐approach goals and leaders’ performance‐approach goals (higher‐level influences) on employee burnout (individual‐level outcome), we bring stringent and long‐needed empirical support for this basic assumption.

### 4.1 Theoretical implications

Our research makes several important contributions. First, although researchers started to investigate the role of individuals’ motivational strivings in relation to burnout (e.g., Naidoo et al., 2012; Poortvliet et al., 2015), our understanding of the role of employees’ direct leaders’ motivational strivings remains limited. While these initial studies suggest that burnout may result from frustrations in one's own goal striving, our multilevel perspective demonstrates that leaders’ dispositional motivation also has a pervasive influence in explaining variance in employee burnout. Thus, on the one hand, we provide further support for a motivational account of burnout by identifying goal strivings as a major antecedent of burnout. On the other hand, we provide nuance to this motivational perspective by showing that not only individual goal strivings but also leaders’ motivational strivings are a significant contributor provoking employee burnout.

Second, our results contribute to the achievement goal literature by providing evidence of cross‐level effects of leaders’ motivational strivings on individual‐level outcomes above and beyond individuals’ motivational strivings. In contrast to the appreciable body of research on achievement goals at the individual level, there has been a lack of progress on how achievement goals at higher levels of analysis (i.e., leaders) affects individual‐level outcomes (for an exception, see Dragoni & Kuenzi, 2012). As such, our results address the call for multilevel research to investigate the cross‐level influence of leaders’ achievement goals in predicting individual‐level outcomes. While research in educational psychology provides support for the influence of group or environmental characteristics (e.g., Murayama & Elliot, 2009; Wolters, 2004), the evidence for such effects is far less understood in other contexts. We provide relatively robust evidence that motivational strivings of leaders critically affect lower‐level outcomes.

Finally, these results strengthen the importance of considering the leader role when it comes to the development of burnout (cf. De Hoogh & Den Hartog, 2009). Our findings position our work centrally within a larger literature that acknowledges leaders’ unique position to influence employees’ work outcomes (e.g., Chen & Bliese, 2002; Dragoni, 2005). Importantly, it adds to the burnout literature by providing empirical evidence demonstrating that environmental factors meaningfully contribute to the explanation of individual‐level outcomes beyond individual‐level perceptions (Leiter & Maslach, 1988). This novel insight in the motivational etiology of burnout may help in inspiring a new series of theory‐driven interventions to prevent and/or combat the development of burnout. Meta‐analytic summaries of organizational stress interventions (Richardson & Rothstein, 2008) show that the large majority of stress interventions are directed toward the individual, with very few studies having looked to address stressors in the immediate work environment. Our research suggests that an exclusive focus on individuals’ motivational traits might be of limited value given the independent effects of leader achievement goals. Thus, developing and testing interventions that focus on the leaders’ motivational profile or their effects might offer alternative ways to understand and prevent potentially stressful dynamics in the work environment.

### 4.2 Limitations and future research directions

Despite a number of strengths, our study also has several limitations. First, the use of cross‐sectional data does not permit us to infer causal flows from leaders’ achievement goals and employees’ achievement goals to employees’ burnout. Although there is a good rationale for top‐down ordering of predictors in multilevel designs (see Mathieu & Taylor, 2007), we cannot rule out the possibility of reverse causality. Note that the cross‐sectional nature of the data may be less of a limitation because of the multisource nature of our data and the separation of measurements in time in Study 2 (Podsakoff et al., 2003).

A second limitation is the use of self‐report measures in the current study. Although the variables in our study were best assessed by directly asking the respondents themselves (measures of achievement goals and burnout), future research should ideally incorporate measures of different sources (e.g., objective reports of withdrawal at work and medical diagnoses of burnout) in order to complement the subjective reporting of feelings of burnout by employees.

A third limitation relates to the sampling procedure in Study 1. First, we lack information about the number of organizations that were contacted by students and that actually participated. Second, leaders’ selection of up to five employees to participate in the survey might have been arbitrary rather than random. Although these sampling procedures limit the generalizability of our findings, the multi‐organizational sample and the use of a different sampling procedure in Study 2 may outweigh these concerns.

Finally, our aim was to investigate the cross‐level direct effects of leaders’ motivational strivings on employee burnout above and beyond employees’ own motivational strivings. However, there may well be cross‐level interactions whereby the relationships between lower‐level predictors and outcomes differ as a function of higher‐level factors. We exploratively examined such cross‐level interaction effects in our sample, but we did not find compelling and consistent evidence for such patterns. For example, future research may test whether the relationship between individuals’ motivational strivings and their feelings of burnout might be affected by leaders’ motivational strivings.

In terms of future research, we see three important avenues. First, we currently have a limited understanding of the mechanisms underlying our effects. While we hinted at some of the processes likely involved (e.g., access to resources), we need an empirical test of these processes. To do so, more conceptual work is needed linking achievement goal theory to the main explanatory models of burnout, such as the job‐demands resources model (Demerouti et al., 2001). Second, we focused on leader achievement goals as a motivational cue in the work environment. However, there are other motivational cues in the work environment that may come into play in the genesis of burnout. For instance, future research may want to look at achievement goals of close coworkers and how these affect an individual's burnout. Furthermore, understanding how team members’ individual achievement goal profiles lead to the emergence of a team achievement goal climate and its role in burnout may be particularly valuable at times when teamwork is a fundamental aspect of organizational life. Third, future research may want to start developing stress interventions that address motivational strivings of leaders. We would not go so far as to suggest that leaders can be identified or selected on the basis of their achievement goals. However, while achievement goals are conceptualized as personality traits, they also have more state‐like aspects that can make them more malleable. For example, by means of a mind‐set training (Heslin, Latham, & VandeWalle, 2005), leaders may be trained to focus more on learning and development aspects, which may be especially beneficial for leaders high in performance‐approach goals.

### 4.3 Practical implications

Given the high organizational and societal costs of burnout, identifying its personal and contextual antecedents is important. Our results suggest that employees’ mastery goals may reduce burnout. More interesting, however, is that motivational strivings of leaders also have direct effects on employee burnout. Organizations may therefore not only intervene on the individual level, but also might shift focus to the leader level. Our findings suggest that organizations that create an environment in which leaders are encouraged to adopt mastery‐approach goals rather than performance‐approach goals may have an advantage in this regard (VandeWalle, 2003). Research indeed has shown that intervention programs that focus on changing environmental factors (e.g., leader's motivational strivings) seem to be effective in reducing the experience of burnout (Halbesleben & Buckley, 2004). Organizations may stimulate mastery‐approach goals in leaders by emphasizing self‐improvement rather than self‐enhancement, and by accepting errors or mistakes as part of the learning process, particularly in training programs (e.g., Ames, 1992; Dragoni, 2005; Van Yperen & Orehek, 2013).

## 5 CONCLUSIONS

Reducing burnout is important for organizations, and leaders play a pivotal role in creating appropriate goal contexts for employees. However, the role of leaders in employees’ burnout has received scant attention (for an exception, see De Hoogh & Den Hartog, 2009). By applying a multilevel perspective, this study extends previous research by showing the importance of leaders’ achievement goals in predicting employee burnout above and beyond employees’ own achievement goals. The cross‐level effects between leaders’ motivational strivings and employee burnout provide an interesting area to enhance our understanding of when leaders’ achievement goals are beneficial or detrimental to employee well‐being and behavior. The current insights should provide an important impetus for further extending our understanding of cross‐level environmental factors in relation to employee burnout.

## CONFLICT OF INTERESTS

The author(s) declared no potential conflicts of interest with respect to the research, authorship, and/or publication of this article.

## ENDNOTES

1 We also tested our results without including leader mastery‐avoidance goals and leader performance‐avoidance goals, and the effects were unchanged.
